# Characterization analysis and polymorphism detection of the porcine *Myd88* gene

**DOI:** 10.1590/S1415-47572009000200015

**Published:** 2009-06-01

**Authors:** Xinyun Li, Huazhen Liu, Shulin Yang, Zhonglin Tang, Yuehui Ma, Mingxing Chu, Kui Li

**Affiliations:** 1 The Key Laboratory of Animal Genetics, Breeding and Reproduction of Ministry of Education of China, Huazhong Agricultural University, WuhanP.R. China; 2 The Key Laboratory for Farm Animal Genetic Resources and Utilization of Ministry of Agriculture of China, Institute of Animal Science, BeijingP.R. China

**Keywords:** * Myd88*, TLR, polymorphism, pig, chromosome mapping

## Abstract

The myeloid differentiation primary response protein 88 (Myd88) is an essential adaptor protein, which mediates in all Toll-like receptor (TLR) members signal transduction, except for *TLR3*. In this study, the 4464 bp genomic sequence of porcine *Myd88* was first isolated, whereupon tissue distribution, chromosome mapping and single nucleotide polymorphism (SNP) were analyzed. Our results revealed that porcine *Myd88* gene, which was located at chromosome 13 linked with marker S0288 (distance = 40 cR; LOD = 8.66), was widely expressed in all the examined tissues. There were 16 potential SNPs in the isolated genome fragment. SNP 797T/C in the first intron was studied, with no significant association being found between the genotype and immune traits in pigs (p > 0.05). The porcine Myd88 protein contained both the death domain (DD) and the Toll/IL-1 receptor domain (TIR). Leu residues, essential for its structure, were the most abundant encountered in the DD. The TIR contained two conserved motifs which may play important roles in the Myd88 function.

## Introduction

Myd88 is an essential cytoplasmic adaptor protein, critical for Toll-like receptor signal transduction. TLRs play very important roles in host immune reaction defense against invading of microbial pathogens (Lemaitre *et al.*, 1996; Medzhitov and Janeway, 1997). So far, 11 TLR (*TLR1-11*) members have been characterized (Hardiman *et al.*, 1996; Medzhitov and Janeway, 1997; Palladino *et al.*, 2007). Myd88 is associated with all TLR signaling pathways except for that of *TLR3* (Li *et al.*, 2005).

The Myd88 protein contains a Toll/IL-1 receptor domain (TIR) in its C-terminus and a death domain (DD) in its N-terminus (Uematsu and Akira, 2006). All TLRs contain TIR in their cytoplasmic domain. On stimulation, TLRs recruit Myd88 through the TIR-TIR interaction. Myd88 recruits downstream molecular IL-1 receptor kinase (*IRAK*) to TLRs through the DD-DD interaction. Four IRAK members (IRAKs) have been identified so far, these being *IRAK1*, *IRAK2*, *IRAK-M* and *IRAK4*. *IRAK1* and *IRAK4* are activated via phosphorylation in response to stimuli. The downstream molecule, tumor necrosis factor receptor-associated factor 6 (TRAF6), is then activated by IRAKs. Subsequently, TRAF6 activates growth factor-β- activated protein kinase 1 (TAK1) in a ubiquitin-dependent manner. Finally, TAK1 activates the IKK complex, which leads to activation of the *NF*-κ*B* transcription factor. This TLR signaling pathway is called the Myd88-dependent pathway (Takeda and Akira, 2004; Yamamoto and Akira, 2004). It is essential for the expression of inflammatory cytokines, including *TNF*α, *IL-6*, *IL-12*, *IL-1*β, as well as co-stimulatory molecules (Adachi *et al.*, 1998; Takeda and Akira, 2004). Inflammatory reactions activated by these inflammatory cytokines are responsible for the removal of invading pathogens, these including bacteria, viruses and protozoans (Adachi *et al.*, 1998; Takeda and Akira, 2004; Yamamoto and Akira, 2004). Previous studies indicated that the expression level or mutations of the *Myd88* gene are related to important phenomena such as endotoxin tolerance (Li *et al.*, 2000; Medvedev *et al.*, 2002). *Myd88* deficient mice present defects in T cell proliferation, thereby lacking in response to IL-1 and IL-18 (Adachi *et al.*, 1998). They also displayed low resistance to protozoan infection (Scanga *et al.*, 2002). Thus, *Myd88* plays very important roles in inflammatory reactions and host defense against infections. Consequently, porcine *Myd88* may be an important candidate gene for disease-resistance breeding.

In this study, we first isolated the genomic DNA sequence of the porcine *Myd88* gene. We then analyzed tissue distribution, chromosome mapping, polymorphisms and structure characterization. We also studied one SNP in the first intron of porcine *Myd88* by the polymerase chain reaction-restriction fragment-length polymorphism (PCR-RFLP) method. Association analysis with pig immune traits indicated that there was no significant association in our experimental group. Our results provide useful information for further studies on the porcine *Myd88* gene.

**Figure 1:**
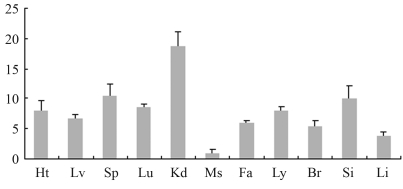
mRNA tissue distribution of the porcine *Myd88* gene assessed by real-time PCR. Error bars represent the SD (n = 3). Relative mRNA expression levels of the *Myd88* gene were normalized by endogenous β-*actin* expression. Ht: heart, Lv: liver, Sp: spleen, Lu: lung, Kd: kidney, Ms: skeletal muscle, Fa: fat, Ly: lymph node, Br: brain, Si: small intestine, Li: large intestine.

**Figure 2:**
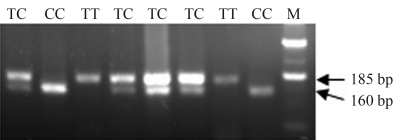
RFLP analysis of porcine *Myd88* gene polymorphism. 797T/C polymorphism was detected by *ApaL I* (TT 185 bp, CC 160/25 bp, TC 185/160/25 bp). M: DNA ladder.

**Figure 3:**
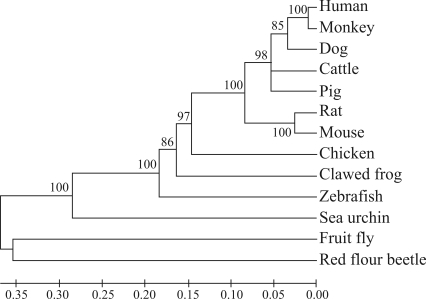
The phylogenetic tree of the *Myd88* gene. Bootstrap confidence values, shown at the nodes of the tree, are based on 1000 bootstrap replicates. Horizontal branch lengths are proportional to the estimated divergence of the sequence from the branch point. GenBank accession numbers are: Human, AAB449967; Monkey, XP_001088062; Dog, XP_534223; Cattle, NP_001014404; Pig, ABM90642; Rat, AAH9726; Mouse, AAC53013; Chicken, NM_001030962; Clawed frog, NP_001016837, Zebrafish, AAQ90476; Sea urchin, XP_780590; Fruit fly, NP_610479; Red flour beetle, XP_973419.

**Figure 4:**
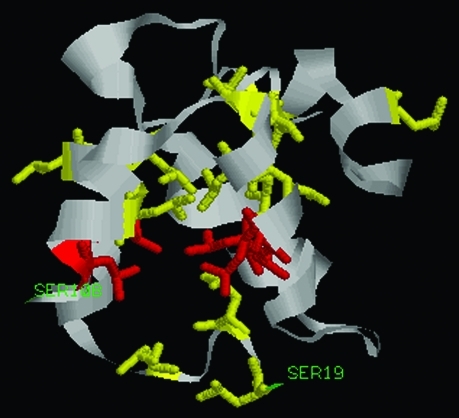
A 3-D model of the porcine Myd88 death domain (DD). The first residue (Ser19) and the last residue (Ser108) are labeled in green. All the Leu residues (labeled in yellow or in red) are displayed in the stick model. The hydrocarbon chains of Leu residues are packed inside, thus forming a hydrophobic interior. The highly conserved Leu residues (Leu33, 75, 89, 90, 105) labeled in red form a Leu plane.

**Figure 5:**
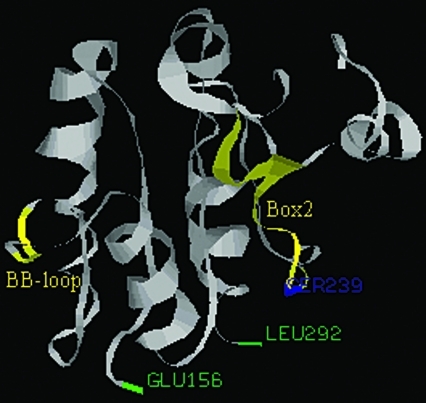
A 3-D model of the Toll/IL-1 receptor domain (TIR) of porcine Myd88. The first residue (Glu156) and the last residue (Leu292) are labeled in green. The highly conserved BB-loop and Box2 are labeled in yellow. The Ser239 residue, the possible phosphorylation site, is labeled in blue.

## Materials and Methods

### Isolation of the porcine *Myd88* gene

For genomic DNA isolation, DNA fragments (TI Nos: 768175941; 773990928; 775670732; 847811271; 853168567; 854250486; 857233111; 861225716; 1420071428; 1420478383; 1420500667) of the porcine *Myd88* gene were retrieved from GenBank through sequence alignment. Primers were then designed according to this sequence information. PCRs were performed for genomic DNA isolation of the porcine *Myd88* gene. PCR profiles were 5 min at 95 °C, followed by 35 cycles of 30 s at 94 °C, 30 s at annealing temperature, 90 s at 72 °C and a final extension of 5 min at 72 °C. All PCR products were sequenced by commercial services.

### Chromosome mapping of the porcine *Myd88* gene

The radiation hybrid (RH) panel was used for porcine *Myd88* gene chromosome mapping analysis (Yerle *et al.*, 1998). PCR was performed in a 10 μL reaction mixture containing 25 ng of cell hybrid line DNA, a 1x PCR buffer (TaKaRa, Dalian, P. R. C), 0.3 μM of each primer (Table 1), 75 μM of each dNTPs, 1.5 mM MgCl_2_ and 1 U Taq DNA polymerase (TaKaRa, Dalian, P. R. C). The PCR profile was 5 min at 94 °C followed by 35 cycles of 30 s at 94 °C, 30 s at 61 °C, 40 s at 72 °C, and a final extension of 5 min at 72 °C. PCR results were analyzed using the IMpRH mapping tool (Milan *et al.*, 2000).

### Tissue distribution of the porcine *Myd88* gene

For tissue distribution analysis, eleven tissues including heart, liver, spleen, lung, kidney, fat, skeletal muscle, lymph node, small intestine, large intestine and brain were obtained from four 18 to 24-months-old Wuzhishan mini-pigs. Total RNA was extracted from each sample using the TRIzol reagent (Invitrogen, San Diego, CA), and then treated with RNase-free DNase I (MBI Fermentas, Germany). RNA concentration was determined, and equal amounts of RNA from each targeted tissue sample from different individuals were mixed to form the RNA pool. The RNA pool from each sample was reverse-transcribed into cDNA by means of M-MLV reverse transcriptase (Promega, USA). Real-time PCR was performed in a 20 μL mixture containing 1x PCR buffer (TaKaRa, Dalian, P. R. C), 3.0 mM MgCl_2_, 100 μM each dNTP, 0.3 μM gene-specific primers (Table 1), 0.3x SYBR Green I, 2 U Taq DNA polymerase (TaKaRa, Dalian, P. R. C), and 2 μL template cDNA. Reactions were carried out in an Opticon 2 real-time cycler (MJ Research, Waltham, MA), the cycling conditions consisting of an initial 5 min at 95 °C, followed by 35 cycles of 15 s at 95 °C (for denaturation), 30 s at 65 °C (for annealing), 30 s at 72 °C (for elongation) and fluorescence acquisition at 83 °C for 1 s. PCR was performed in triplicate and gene expression levels were quantified relative to the expression of endogenous β-*actin*, by using Gene Expression Macro software (Bio-Rad, Richmond, CA) employing an optimized comparative Ct (ΔΔCt) value method. Expression was considered undetectable when the Ct value of the targeted gene exceeded 35.

### SNPs identification and association analysis

DNA samples from seven breeds, including three Chinese indigenous (Wuzhishan, Laiwu, Bamaxiang and Guizhouxiang) and two foreign breeds (Landrace and Yorkshire), were used as PCR templates for *Myd88* genomic DNA isolation. All PCR products were sequenced. Subsequently, all sequenced information related to the porcine *Myd88* gene, this including our PCR results, the ESTs and genomic DNA fragments available on NCBI, was used to analyse potential SNPs. A potential SNP site was considered as that where different alleles appeared more than twice.

Genetic variation was studied in seven unrelated breeds of pigs, namely, Tongcheng, Wuzhishan, Laiwu, Bamaxing, Guizhouxiang, Yokshire and Landrace. The experimental group underwent association analysis. This group consisted of three pure-blood populations, Tongcheng (T), Landrace (L) and Yorkshire (Y), and two crossbred populations, LYT (L male x YT female) and YLT (Y male x LT female). Six porcine immune-traits were examined. These were red blood cell count (RBC), hematocrit (HCT), mean corpuscular volume (MCV), IgG, blood cell distribution width (RDW) and delayed-type hypersensitivity (DTH). In order to determine immune-traits, the blood from 20-weeks-old pigs was collected so as to detect RBC, HCT, MCV and RDW, by using a blood cell auto-analyzer (MEK-5216K). IgG concentration was ascertained through the radial immuno-diffusion method. The Delayed-type hypersensitivity (DTH) trait was detected by means of the phytohemagglutinin (PHA) skin test, according to the van Heugten method, with a minor modification (Van Heugten *et al.*, 1994).

A general linear model (GLM) was used to estimate the association between genotypes and immune traits. According to the structure of the population, the model used for trait association analysis is described as follows:

*Y*_*ijk*_ = μ + *P*_*i*_ + *G*_*j*_ + *B*_*k*_ + (*PG*)_*ij*_ + (*PB*)_*ik*_ + (*GB*)_*jk*_ + *e*_*ijk*_

where *Y*_*ijkl*_ = *l*^th^ trait measured in the animal; μ = overall mean; *P*_*i*_ = fixed effect of the *i*^th^ population (*i* = 1, 2, 3, 4, 5); *G*_*j*_ = fixed effect of the *j*^th^ genotype (*j* = 1, 2, 3); *B*_*k*_ = fixed effect of the *k*^th^ batch (phenotypic data were recorded in two periods, *k* = 1, 2); (*PG*)_*ij*_ = effect of interaction *i*^th^ population *j*^th^ genotype; (*PB*)_*ik*_ = effect of interaction *i*^th^ population *k*^th^ batch; (*GB*)_*jk*_ = effect of interaction *j*^th^ genotype *k*^th^ batch; *e*_*ijkl*_ = error term.

### Phylogenetic tree and structure analysis

Myd88 proteins from many species were collected for phylogenetic tree analysis and the homologues of the sequences analyzed by means of the ClustalW program. A phylogenetic tree was retrieved by using MEGA 3.1 software. Conserved residues of the functional domain of porcine Myd88 were analyzed through multiple sequences alignment. The three-dimensional (3-D) model was predicted through the 3djigsaw program. An image of the 3-D model was obtained by using software Raswin 2.7 software. Phosphorylation sites were predicted by the NetPhos program.

## Results and Discussions

### Isolation and chromosome assignment

The DNA segment isolated was 4464 bp, subsequently deposited in to GenBank (GenBank no, EU056737). The isolated genomic sequence contained the complete ORF (882 bp) of the porcine *Myd88* gene (NM_001099923). RH mapping results revealed that this gene was assigned to the long arm of the pig chromosome 13 (SS13q), the closest linked marker being S0288 (distance = 40 cR; LOD = 8.66). In humans, *Myd88* has been mapped on 3p22 (Bonnert *et al.*, 1997). Comparative genomic analysis results confirmed that pig chromosome 13 is homologous with human chromosome 3 (Sun *et al.*, 1999). Thus, our mapping results conformed to those from comparative genomic analysis.

### Detection of tissue distribution

Real time-PCR analysis was performed to determine the mRNA expression profile of the *Myd88* gene in Wuzhishan mini-pigs. The data revealed *Myd88* gene expression in all examined tissues, this expression being relatively low in skeletal muscle tissue (Figure 1). Previous studies also showed wide *Myd88* gene expression in digestive tissues, the spleen and mesenteric lymph nodes (Tohno *et al.*, 2007). In humans, *Myd88* was found to be constitutively expressed in many tissues (Hardiman *et al.*, 1996), this thus implying the similarity of the tissue distribution profile of the porcine *Myd88* gene to that in humans. TLRs are widely expressed in many tissues (Zarember and Godowski, 2002), and Myd88 functions as the adaptor protein of TLRs. Moreover, sub-cellular localization results confirmed that Myd88 found in cytoplasm was not a secretory protein (Nishiya *et al.*, 2007). Therefore, *Myd88* needs to be widely expressed in order to participate in TLR signal transduction.

### Polymorphism detection and association analysis

Porcine *Myd88* gene polymorphisms were detected by multiple sequence comparison. According to our results, the 4464 bp genomic DNA of *Myd88* contained 16 potential SNPs which were 797T/C, 813A/g, 1721T/g, 1755C/A, 2130T/A, 2461C/T, 2468G/A, 2519G/A, 2743A/g, 2757C/T, 3076A/g, 3258A/g, 3291T/A, 3298C/T, 3345A/g and 3485G/A. None of these resulted in residual changes, this indicating that the porcine Myd88 protein was highly conserved. 797T/C polymorphism of the porcine *Myd88* gene, which can be detected by the PCR-RFLP method, was further studied. The genotypes of this site were identified by using the restriction enzyme *Apa*L I (TT 185 bp, CC 160/25 bp, TC 185/160/25 bp) (Figure 2). Allele frequency analysis revealed a much higher frequency of allele T in five Chinese indigenous breeds than in Landrace and Yorkshire (Table 2). We performed a preliminary association study to determine whether this polymorphism had affected any immune-traits in the pig. The data showed that there was no significant association between this SNP and immune traits RBC, HCT, MCV, IgG, DTH and RDW (p > 0.05) (Table 3).

### Phylogenetic tree and structural characterization

The porcine Myd88 protein contained 293 residues with an overall sequence similarity to Myd88 in human (88%), chicken (70%), clawed frog (65%), zebrafish (62%) and sea urchin (40%). Phylogenetic tree analysis also showed that Myd88 was conserved during evolution (Figure 3). Highly conserved residues of Myd88 were detected through sequence comparison with the five species mentioned above. Porcine Myd88 contained two functional domains, DD (residues: 19-109) and TIR (residues: 157-293) (Tohno *et al.*, 2007), the most abundant amino acid in DD being Leu (19.8%). 17 DD residues were conserved in all the species examined. Among the conserved residues, there were 7 Leu residues and 12 hydrophobic residues (A, I, L, F, W, and V belong to hydrophobic amino acid). These results indicate that hydrophobic residues, especially of Leu, may play important roles in maintaining DD structure and functioning.. The 3-D model of the porcine Myd88 death domain was predicted using the 3djigsaw program. According to the model, the hydrocarbon chains of Leu residues were packed in the inner part of the DD, thus forming a hydrophobic interior. The highly conserved Leu residues (Leu33, 75, 89, 90, 105) formed a Leu plane (Figure 4). These results also indicate that Leu residues may play important roles in the death domain.

The other functional domain of porcine Myd88 is a TIR containing 137 residues. A BB-loop, which was found in the TIR domain of TLRs (Xu *et al.*, 2000), was also found in the TIR of the porcine Myd88 in our studies. This loop contained the motif (RDxLPG, x represents L or V), and was found to be highly conserved among all the species studied. Previous research has confirmed that the BB-loop was essential for maintaining theTLR4 function. Substitution of the Pro residue in the BB-loop of TLR4 by His abolished the *TLR4* immune-response to lipopolysaccharide (Poltorak *et al.*, 1998). The BB-loop, highly conserved during evolution, may be very important for the porcine Myd88 signaling pathway. In addition, another conserved motif (CDFQTKFAxSL, x represents L or V) was found in the TIR of porcine Myd88 (Box 2). This motif contains a conserved Ser which may be a phosphorylation site predicted through using the NetPhos program (p = 0.959), and may be related to phosphorylation of porcine Myd88. A 3-D model of the TIR of porcine Myd88 was predicted by using the 3djigsaw program (Figure 5), the conserved domains being labeled in yellow and the Ser in Box 2 in blue.

## Figures and Tables

**Table 1:** Primers used for porcine *Myd88* isolation, SNPs detection and mRNA tissue distribution analysis.

Gene	Primers	5'-3' sequence	TM (°C)	Length (bp)
*Myd88*	DNAPL1	GGAAGCACAGGCCCACAAG	60	1380
	DNAPR1	GGTGATGCCTGACATCCAAG		
	DNAPL2	AGCACGAGGCAGCTGAGAAG	61	1308
	DNAPR2	TTGGTGCAGGGGTTGGTGTAG		
	DNAPL3	CGGATGGTAGTGGTTGTCTCTGA	62	1491
	DNAPR3	CCTGTATAAGCGTCTCTGCGTG		
	DNAPL4	CCCAACTTCTGACATCTCCATC	61	1046
	DNAPR4	CCTGCTAAGTTTGGTTCCTGTG		
	MapPL	CCCAACTTCTGACATCTCCATC	61	1046
	MapPR	CCTGCTAAGTTTGGTTCCTGTG		
	QpcrPL	CGGATGGTAGTGGTTGTCTCTGA	65	194
	QpcrPR	TTGGTGCAGGGGTTGGTGTAG		
	SNPPL	AAAAATTCCTCAGGTTCCTAGAAAGTGCA	60	185
	SNPPR	GATCCACAGCTGATGTGAGCA		

β-*actin*	ControlPL	GGACTTCGAGCAGGAGATGG	65	233
	ControlPR	GCACCGTGTTGGCGTAGAGG		

**Table 2:** Genotypes and allelic frequencies for the polymorphism 797 T/C of *Myd88* in several pig breeds

Breeds	N	Genotypes				Allele frequencies	
		TT	TC	CC		T%	C%
Wuzhishan	35	35	0	0		100	0
Bamaxiang	33	33	0	0		100	0
Guizhouxiang	38	38	0	0		100	0
Tongcheng	42	40	1	1		96.4	3.6
Laiwu	37	19	18	0		75.7	24.3
Yorkshire	38	14	17	7		59.2	40.8
Landrace	31	7	14	10		45.2	54.8

**Table 3:** Association analysis of 797 T/C polymorphism in *Myd88* with porcine RBC, HCT, MCV, IgG, DHA and RDW traits.

Genotypes	N	RBC	HCT	MCV	IgG	DTH	RDW
CC	24	6.34 ± 0.32	37.32 ± 2.02	59.20 ± 1.40	50.50 ± 3.27	8.73 ± 0.39	18.12 ± 0.42
TC	65	6.57 ± 0.29	37.26 ± 1.83	56.11 ± 1.27	43.95 ± 2.96	8.96 ± 0.35	18.85 ± 0.38
TT	68	6.61 ± 0.19	37.64 ± 1.18	56.37 ± 0.82	45.44 ± 1.92	8.76 ± 0.23	18.46 ± 0.25
p-value*		0.777	0.984	0.171	0.274	0.876	0.402

*Means the probability of F-test for the genotype effect. Phenotypic value = mean ± SE. RBC, red blood cell count; HCT, hematocrit; MCV, mean corpuscular volume; IgG, immunoglobulin G; DTH, delayed-type hypersensitivity; RDW, blood cell distribution width.
